# Emergence of Flucytosine-Resistant *Candida tropicalis* Clade, the Netherlands

**DOI:** 10.3201/eid3107.241918

**Published:** 2025-07

**Authors:** Fatima Zohra Delma, Bram Spruijtenburg, Jacques F. Meis, Auke W. de Jong, James Groot, Johanna Rhodes, Willem J.G. Melchers, Paul E. Verweij, Theun de Groot, Eelco F.J. Meijer, Jochem B. Buil

**Affiliations:** Radboud University Medical Center, Nijmegen, the Netherlands (F.Z. Delma, B. Spruijtenburg, J.F. Meis, J. Rhodes, W.J.G. Melchers, P.E. Verweij, T. de Groot, E.F.J. Meijer, J.B. Buil); Canisius-Wilhelmina Hospital (CWZ)/Dicoon, Nijmegen (B. Spruijtenburg, T. de Groot, E.F.J. Meijer); University of Cologne, Cologne, Germany (J.F. Meis); National Institute for Public Health and the Environment (RIVM), Bilthoven, the Netherlands (A.W. de Jong, J. Groot, P.E. Verweij)

**Keywords:** *Candida*, *Candida tropicalis*, flucytosine, antifungal resistance, genotyping, whole-genome sequencing, antimicrobial resistance, fungi, the Netherlands

## Abstract

*Candida tropicalis* is the second most virulent *Candida* species after *C. albicans*. Previous studies from the Netherlands and France reported a notable reduction in susceptibility to flucytosine (5-FC) in a substantial proportion of *C. tropicalis* isolates. We investigated epidemiologic patterns of *C. tropicalis* isolates in the Netherlands and the genetic mechanisms driving widespread non–wild-type (WT) 5-FC resistance. We conducted antifungal susceptibility testing and used advanced molecular techniques, including short tandem repeat genotyping and whole-genome sequencing paired with single-nucleotide polymorphism analysis, to analyze 250 C. *tropicalis* isolates collected across the Netherlands during 2012–2022. Our findings revealed the rapid emergence of a 5-FC–resistant, non-WT *C. tropicalis* clade, accounting for >40% of all *C. tropicalis* isolates by 2022. Genomic analysis identified a homozygous nonsense mutation in the *FCY2* gene, which was exclusive to this non-WT population. Continued surveillance efforts are needed to detect and prevent the spread of drug-resistant *Candida* species.

*Candida tropicalis* is among the 5 most common *Candida* species found in healthcare settings (*1*–*3*). This diploid yeast is prevalent in Latin America and Asia and is occasionally reported in Africa and Europe (*3*,*4*). Since the 2000s, *C. tropicalis* has emerged as a substantial cause of candidemia, particularly in patients with neutropenia (*5*). *C. tropicalis* is considered the second most virulent *Candida* species, after *C. albicans* (*6*). Antifungal drug resistance has been increasingly reported for *C. tropicalis*, especially resistance to azoles (*7*,*8*) and, to a lesser degree, amphotericin B and echinocandins (*9*,*10*). The World Health Organization lists *C. tropicalis* as a high-risk pathogen (*11*,*12*), underscoring its considerable threat to public health and the need for research and surveillance.

Echinocandins are the first-line treatment for candidemia. Flucytosine, also known as 5-fluorocytosine (5-FC), is used to treat severe invasive candidiasis, which can cause endocarditis, endophthalmitis, or meningitis (*13*,*14*). Although 5-FC is only used in combination therapy because of the rapid emergence of isolates with high MICs (*14*), it shows potent activity against most yeasts, including *C. tropicalis* (*15*). Global rates of 5-FC–resistant, non–wild-type (WT) *C. tropicalis* are low, at ≈10% (*15*,*16*). However, we previously found a high percentage of *C. tropicalis* isolates with increased 5-FC MICs in the Netherlands (*17*), which has also been observed in France, where susceptibility to 5-FC has been documented in non-WT *C. tropicalis* since the 1980s (*18*). A 4-year survey conducted during 2002–2006 in the Paris area revealed increased 5-FC MICs in 45 (35%) of 130 *C. tropicalis* isolates recovered from blood cultures. Specific genetic mutations in the *URA3* gene were observed in all isolates with increased 5-FC MICs. In addition, the non-WT strains shared identical multilocus sequence typing (MLST) genotypes, indicating clonal spread (*18*).

To investigate the recent decrease in 5-FC susceptibility in *C. tropicalis* isolates in the Netherlands, we performed a literature review and used available epidemiologic data from the Radboud University Medical Center CWZ Center of Expertise for Mycology (Nijmegen, the Netherlands). We applied a newly developed, highly reproducible short tandem repeat (STR) assay and whole-genome sequencing (WGS) to genotype *C. tropicalis* isolates (*19*,*20*), describe the epidemiology of *C. tropicalis* isolates over time, and identify the genetic basis of the non-WT 5-FC–resistant phenotype. Because the data consisted solely of information about clinical strains and did not include patient details, no ethics approval was required according to local guidelines.

## Materials and Methods

### Literature Review

To obtain an updated overview of 5-FC resistance in *C. tropicalis*, we conducted a comprehensive literature search across electronic databases, including PubMed and Google Scholar, by using the keywords “*C. tropicalis*,” “5-FC/flucytosine/fluorocytosine resistance,” and “clonal resistance in *C. tropicalis*.” We also reviewed citations within the retrieved studies. We identified 15 relevant studies and extracted data on geographic region, time period, number of isolates, antifungal susceptibility testing (AFST) methods, interpretation criteria, and rates of non-WT 5-FC resistance.

### Clinical Isolate Collection

A total of 250 nonreplicated clinical isolates of *C. tropicalis* were collected from patients across the Netherlands during January 2012–May 2022. The sources of isolates were as follows: blood, other sterile sites, oropharynx (including sputum and bronchoalveolar lavage samples), vagina, feces, urine, and other superficial sources. We identified *Candida* spp. by using matrix-assisted laser desorption/ionization time-of-flight mass spectrometry (Bruker, https://www.bruker.com). We stored isolates at −70°C in 10% glycerol and grew them on Sabouraud dextrose agar plates at 30°C for 2–5 days before testing.

### Antifungal Susceptibility Testing

We determined MICs for 5-FC and 9 other antifungal drugs (fluconazole, voriconazole, itraconazole, posaconazole, miconazole, amphotericin B, anidulafungin, caspofungin, and micafungin) according to the European Committee on Antimicrobial Susceptibility Testing (EUCAST) E.def 7.4 microdilution method (*21*). We established local epidemiologic cutoffs (ECOFFs) for 5-FC by using the eyeballing method (*17*) and classified isolates with a 5-FC MIC of >0.5 mg/L local ECOFF as non-WT. For fluconazole, we classified isolates with MICs >1 mg/L ECOFF as non-WT. We defined resistance by using EUCAST breakpoints (version 5.0) as follows: fluconazole, >4 mg/L; itraconazole, >0.125 mg/L; voriconazole, >0.25 mg/L; posaconazole, >0.06 mg/L; anidulafungin, >0.06 mg/L; micafungin, >0.06 mg/L; and amphotericin B, >1 mg/L.

### DNA Extraction and STR Genotyping

We extracted DNA from the isolates after 24 hours of incubation on Sabouraud agar. We suspended single colonies in 1 mL of distilled water in a microcentrifuge tube and extracted DNA by using the High Pure PCR Template Preparation Kit (Roche Diagnostics, https://www.roche.com) according to manufacturer instructions. We genotyped the isolates by PCR amplifying and analyzing 6 STR markers, as previously described (*20*).

### WGS and Single-Nucleotide Polymorphism Analysis

We selected 16 *C. tropicalis* isolates for WGS, including five 5-FC–resistant, non-WT isolates that clustered in 1 clade according to STR genotyping, 3 non-WT isolates that grouped outside the clade, and 8 phenotypically WT isolates. We extracted DNA by using InstaGene Matrix (Bio-Rad Laboratories, https://www.bio-rad.com) and sequenced by using Illumina technology (Illumina, https://www.illumina.com). Initially, we added 200 µL of InstaGene Matrix to the yeast pellets, vortexed at 500 rpm, and incubated for 30 minutes at 56°C, followed by another 30 minutes at 99°C. We then transferred samples to tubes containing glass beads with a particle size of <106 μm (Sigma Aldrich, https://www.sigmaaldrich.com) and conducted 2 rounds of bead beating at 17,000 rpm by using a MagNA Lyser (Roche Diagnostics). We assessed DNA integrity by using a TapeStation 2200 system (Agilent, https://www.agilent.com) and measured DNA concentrations by using a Qubit fluorometer (Thermo Fisher Scientific, https://www.thermofisher.com). We prepared libraries by using the Nextera DNA Flex kit (Illumina) following the manufacturer’s instructions. We performed paired-end, 2 × 150-bp mode sequencing on an Illumina NextSeq 550 system (Illumina).

We compared isolate sequences to *C. tropicalis* sequences retrieved from the National Center for Biotechnology Information Sequence Read Archive (SRA) database (https://www.ncbi.nlm.nih.gov/sra) (Appendix Table 1). We performed WGS single-nucleotide polymorphism (SNP) analysis by using Illumina reads, as previously described (*19*). We aligned reads with the *C. tropicalis* reference genome MYA-3404 (SRA accession no. GCA_013177555.1) by using BWA-MEM version 0.7.17 (https://github.com/j-levy/bwa) and subsequently filtered to remove duplicates, unpaired reads, and reads with MAPQ scores <60. We detected SNPs by using FreeBayes version 1.3.6 (https://github.com/freebayes/freebayes) and removed SNPs with a read depth of <25, quality of <100, allele frequency between 0.15 × depth and 0.45 × depth, and allele frequency between 0.55 × depth and 0.9 × depth. We performed phylogenetic analysis by using VCF2PopTree (https://github.com/sansubs/vcf2pop), MEGA11 version 11.0.10 (https://www.megasoftware.net), and iTOL version 6 (https://itol.embl.de) for visualization. We located the resistance-associated genes *FUR1* (GenBank accession no. EU327981.1), *FCY1* (accession no. EU327982.1), *FCY2* (accession no. HQ166001.1), and *URA3* (accession no. EU288195.1) within the reference genome MYA-3404 and inspected those for missense mutations by using IGV version 2.17.3 (*22*). We assessed copy number variation and large-scale deletions by using YMAP (*23*) for all *C. tropicalis* assemblies, as well as for the MYA-3404 reference strain.

### *URA3* Gene Sequencing

We investigated whether the mechanism underlying 5-FC resistance was related to the *URA3* gene mutation resulting in a K177E amino acid substitution (*18*). We sequenced the *URA3* gene from six 5-FC WT (susceptible) and 24 randomly selected 5-FC–resistant non-WT *C. tropicalis* isolates. We grew the isolates on yeast extract/peptone/dextrose agar plates for 24 hours at 30°C and used a standard DNA extraction protocol (*24*). We transferred cells to 1.5-mL tubes containing 600 µL glass beads (diameter 0.4–0.6 mm) and 250 µL breaking buffer (2% Triton X-100, 1% sodium dodecyl sulfate, 2 mol/L NaCl, 1 mol/L Tris-HCl pH 8, 0.5 mol/L EDTA pH 8, and Milli-Q water), shook the tubes for 30 minutes at 60°C, and centrifuged them at 1,000 × *g*. After centrifugation, we added 700 µL of phenol/chloroform/isoamyl alcohol and shook the mixture for 5 minutes at room temperature. We centrifuged the tubes at 10,000 × *g* for 5 minutes and then transferred the resulting upper layer to a new tube and stored at 20°C (room temperature) until analysis. We amplified the *URA3* gene by using the PCR primers and methods described previously (*25*). All sequence data generated in this study were deposited in the SRA database (BioProject accession nos. PRJNA1090665, PRJNA110750, PRJNA1107503). 

### Epidemiologic Analysis and Data Analysis

We analyzed epidemiologic data from the Radboud University Medical Center laboratory information system to assess the distribution of 5-FC non-WT isolates over time and evaluated temporal trends. We plotted the annual rates of 5-FC non-WT isolates and performed linear regression analysis by using GraphPad Prism (GraphPad, https://www.graphpad.com). We considered the trend to be significant if the slope deviated from zero (p<0.05). We also examined correlations between 5-FC and fluconazole resistance.

## Results

### Literature Review

We retrieved 15 studies describing *C. tropicalis* resistance to 5-FC and summarized those data (Table). Most (n = 12) studies were published during 2000–2012. Data on *C. tropicalis* have been reported from various regions, including global collections and countries, such as the United States, United Kingdom, Japan, South Korea, France, Italy, Spain, and Germany. The Clinical and Laboratory Standards Institute broth microdilution method (*38*) was used predominantly (8 studies) to test *C. tropicalis* isolates, followed by the EUCAST reference broth microdilution method, and then E-test gradient strips, ATB Fungus, and VITEK automated susceptibility testing (all bioMérieux, https://www.biomerieux.com).

**Table:** Reported 5-FC resistance rates in different published reports in study of non–wild-type *Candida tropicalis* clade, the Netherlands*

Total no. isolates	Year collected	Body site of isolation	Methods used†	Country	MIC criteria,‡ mg/L	No. (%) isolates with elevated 5-FC MICs	Reference
70	NA	NA	NA	France	NA	NA (80)	(*26*)
60	NA	Different sites	Disc diffusion test, macrodilution with YNBG broth	United Kingdom	I, 2–8	6 (10)	(*27*)
					R, >8	17 (28)	
30	NA	NA	NCCLS	Italy	I, 8–16; R, >32	0	(*28*)
117	1998–2000	Different sites	NCCLS	Spain	I, 8–16	6 (5.1)	(*29*)
					R, >32	0	
759	1992–2001	Different sites	NCCLS	Worldwide collection§	I, 8–16	60 (1)	(*16*)
					R, >32	NA (7)	
33	2000–2001	Blood	CLSI M27-A2	United States	I, 8–16	0	(*30*)
					R, >32	1 (3)	
34	NA	NA	ATB Fungus	Spain	R, >32	3 (8.8)	(*31*)
62	2001–2002	Blood	NCCLS M27-A2	Japan	R, >32	NA (8.1)	(*32*)
60	2004–2006	Different sites	CLSI M27-A2, Etest	Germany	R, >32	NA (58.3)	(*33*)
130	2002–2006	Blood	EUCAST	France	R, >8	45 (35)	(*18*)
97	2006	NA	Etest	Taiwan	I, 8–16	1 (1)	(*34*)
					R, >32	NA	
303	NA	Blood	CLSI M27-A3	United States	I, 8–16	5 (1.65)	(*35*)
					R, >32	4 (1.32)	
149	2007–2008	Blood	VITEK-2	South Korea	I, 8–16; R, >32	0	(*36*)
126	NA	Blood	CLSI M27-A3	Worldwide collection¶	NA	NA (10.3)	(*37*)
359	2008–2024	Different sites	EUCAST	The Netherlands	>16	106 (29.5)	(*17*)

*5-FC, flucytosine; CLSI, Clinical and Laboratory Standards Institute; EUCAST, European Committee on Antimicrobial Susceptibility Testing; I, intermediate resistance; NA, not applicable; NCCLS, National Committee for Clinical Laboratory Standards; R, resistant; YNBG, yeast nitrogen base glucose. †CLSI was formerly known as NCCLS. CLSI M27 broth dilution method for yeast (*38*). ATB Fungus test, Etest, and VITEK-2 were purchased from bioMérieux (https://www.biomerieux.com). ‡Resistance status and MICs used to interpret level of resistance to 5-FC in each study. §North America, Latin America, Europe, and Asia-Pacific. ¶United States, Europe, Latin America, and the Asia-Pacific region.

The frequency of *C. tropicalis* isolates with elevated MICs to 5-FC ranged from 0%–10% (Italy, Spain, South Korea) to 10%–30% (including studies from Spain, United States, and United Kingdom) in global collections and was >30% in France and Germany. A genetic survey of non-WT *C. tropicalis* isolates from blood samples in Paris, collected during 2002–2006, identified a group of non-WT isolates with the same MLST profile, all having a *URA3^K177E^* mutation (*18*). Epidemiologic analysis indicated that the group of non-WT clones frequently caused candidemia in patients with malignancies and was associated with better outcomes; recurrent spread was noted during the study period. Genetic relatedness of 5-FC non-WT isolates from specific clades in the United Kingdom (2002–2003) and Belgium (1998) has also been suggested (*39*). The spread of the clade from Paris to other regions of France and other countries in Europe has not been investigated further.

### Antifungal Drug Susceptibility Testing

**Figure 1:**
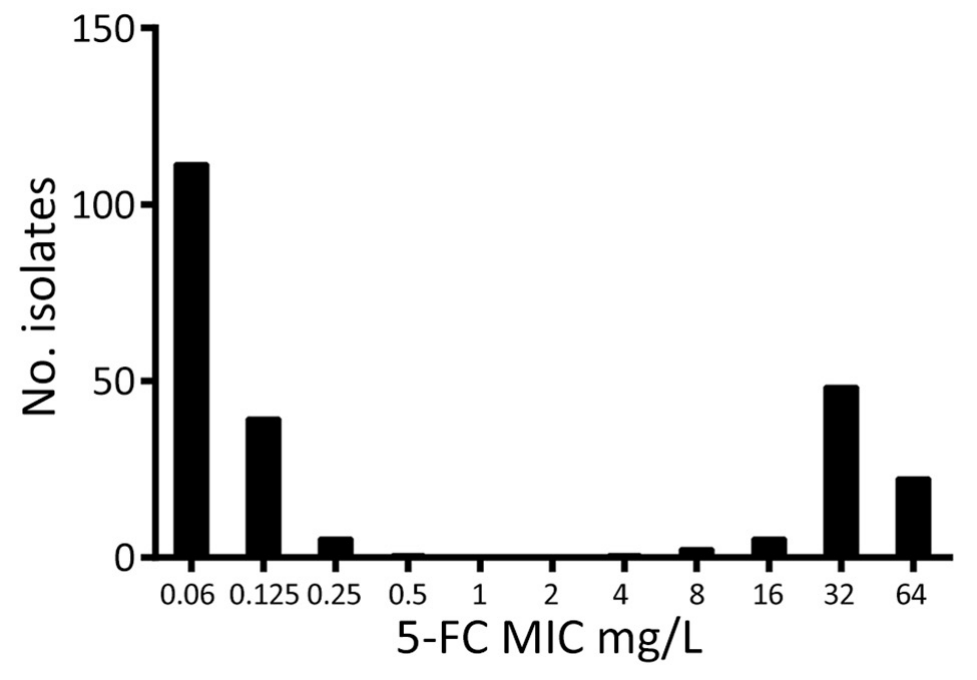
5-FC MIC distribution for 250 *Candida tropicalis* isolates collected in the Netherlands in study of emerging 5-FC–resistant clade. MICs were determined according to European Committee on Antimicrobial Susceptibility Testing microdilution method (*21*). 5-FC, flucytosine.

To gain more insight into the susceptibility of *C. tropicalis* isolates to 5-FC in the Netherlands, we performed AFST on 250 clinical strains, for which the isolation source of 104 isolates was available, by using the EUCAST microbroth dilution (Appendix Table 2). The modal 5-FC MIC was 0.06 mg/L, and local ECOFF was 0.5 mg/L (Figure 1). *C. tropicalis* isolates displayed a bimodal distribution; we observed a clear separation of 2 subpopulations. The first subpopulation consisted of 168 (67.2%) isolates with low 5-FC MICs (<0.5 mg/L) and were classified as WT, whereas the second subpopulation consisted of 82 (32.8%) isolates with high 5-FC MICs (>0.5 mg/L) and were classified as non-WT. AFST of 9 additional antifungal agents did not show that typical bimodal distribution (Appendix Table 3). All isolates were susceptible to amphotericin B. Resistance rates (EUCAST clinical breakpoints) were as follows: fluconazole, 11.3% (28/248); itraconazole, 7.3% (7/95); voriconazole, 8.8% (22/249); posaconazole, 7.7% (4/52); and anidulafungin, 0.8% (2/246). We did not interpret MIC data for miconazole, caspofungin, and micafungin because of the lack of EUCAST clinical breakpoints for those drugs. Overall, 25 (10%) isolates exhibited cross-resistance. We did not observe a correlation between 5-FC non-WT and azole resistance.

### Phylogenetic Analysis Using STR

**Figure 2:**
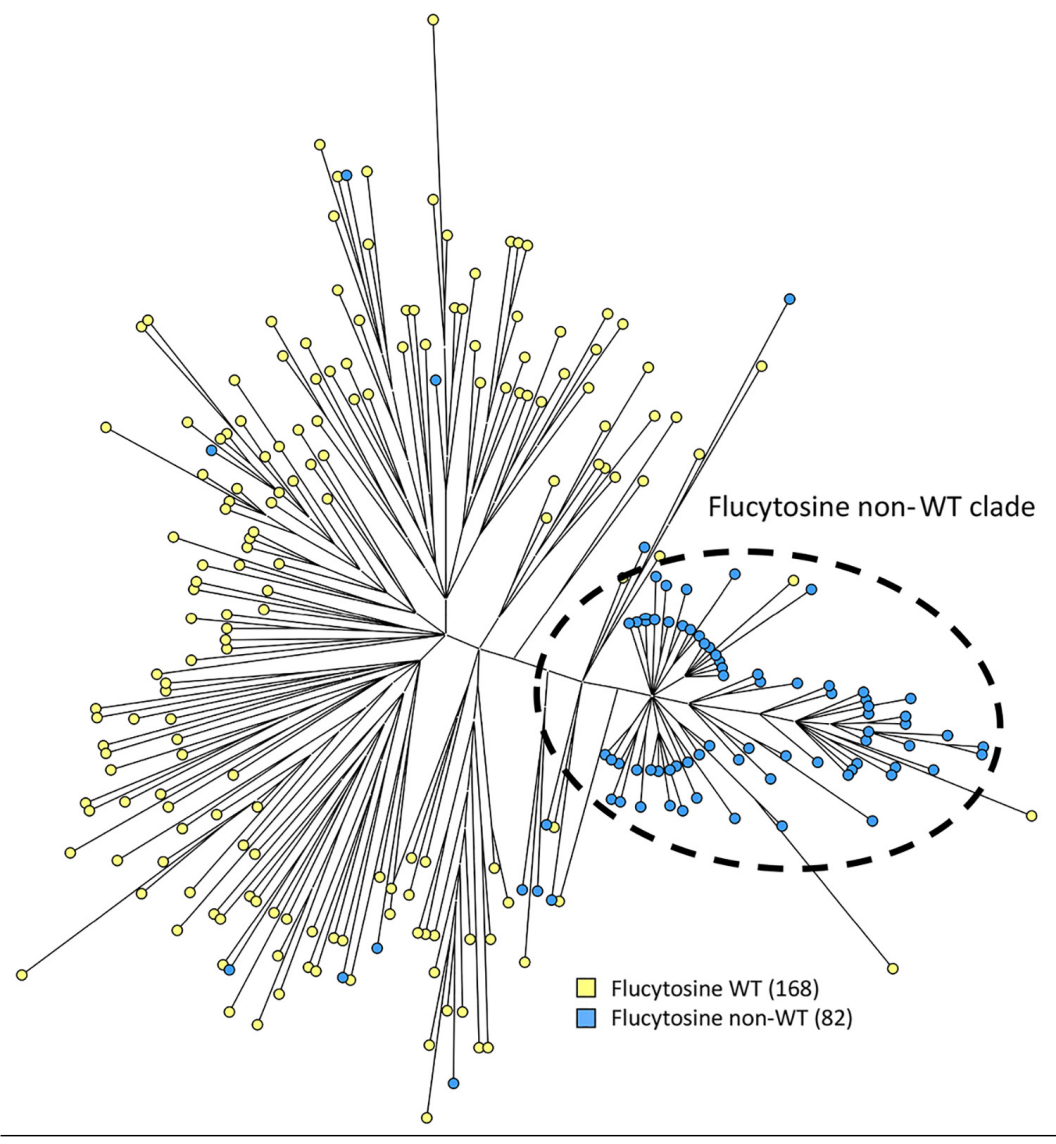
Phylogenetic analysis of *Candida tropicalis* isolates in study of emerging 5-FC–resistant clade, the Netherlands. Maximum parsimony tree of 250 *C*. *tropicalis* isolates was constructed. Colors indicate the number of isolates per flucytosine interpretive category. Isolates with a MIC of <0.5 mg/L were classified as WT and >0.5mg/L as non-WT. WT, wild-type.

We performed STR genotyping for all 250 isolates. Except for 2 isolates (nos. v139–74 and v267–58), which had identical genotypes, all isolates displayed a unique genotype (Appendix Figure). Of the 82 isolates with the 5-FC non-WT phenotype, 65 (79.3%) were closely related and formed a distinct clade, whereas the remaining non-WT isolates did not cluster (Figure 2). Isolates within this clade differed by <4 STR markers; high variability occurred in the first and second markers of the PCR M6 panel, and little variation occurred in PCR M3a and M3b panels (Appendix Figure) (*20*). In contrast, isolates outside this clade differed in >4 markers, usually 5 or 6, and had larger copy number differences. Non-WT strains within the distinct clade containing genotypes 84–147 were isolated during the entire study period.

### WGS Analysis

**Figure 3:**
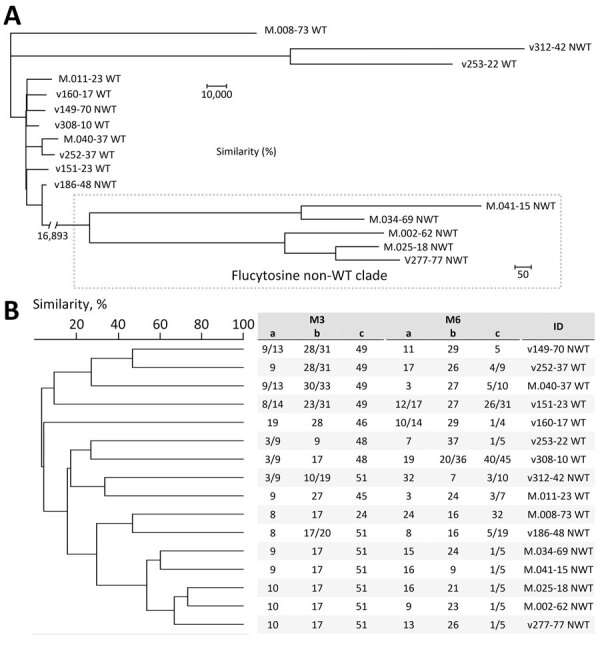
Phylogenetic comparison of relatedness of 16 *Candida tropicalis* isolates in study of flucytosine-resistant *C*. *tropicalis* clade, the Netherlands. A) Tree was constructed by using SNPs identified by whole-genome sequencing. Number on left side below tree indicates number of SNPs for that branch. Scale bars indicate number of SNPs. B) Similarities were determined by short tandem repeat genotyping. M3 and M6 a, b, and c indicate the PCR panel used for genotyping (*20*). Numbers under each PCR panel indicate copy numbers of short tandem repeats for that specific locus. Single numbers indicate homozygous copy numbers; 2 numbers separated by slash indicate heterozygous copy numbers. Flucytosine interpretive category is indicated after the isolate identification number; a MIC of <0.5 mg/L was classified as WT and >0.5 mg/L as non-WT. ID, identification; NWT, non–wild-type; SNP, single-nucleotide polymorphism; WT, wild-type.

**Figure 4:**
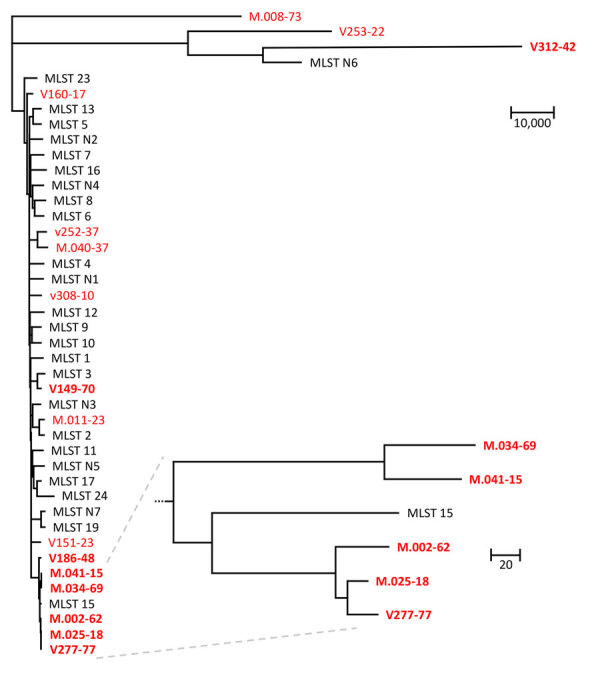
Phylogenetic analysis of global collection of *Candida tropicalis* isolates and 16 isolates from the Netherlands in study of flucytosine-resistant clade. Tree was constructed to compare SNPs. MLST numbers at branch tips correspond to clades. Bold font indicates isolates were flucytosine non–wild-type; red font indicates isolates originating in the Netherlands. Zoomed-in part of tree indicates the five 5-FC non-WT isolates from the Netherlands most closely related to *C. tropicalis* MSLT 15. Scale bars indicate number of SNPs. MLST, multilocus sequence typing; SNP, single-nucleotide polymorphism.

To validate the inferred genetic relatedness, STR outcomes were compared with WGS SNP calling. Isolates that were part of the non-WT clade, according to the STR data (n = 5), also clustered according to WGS SNP analysis (Figure 3). Within the non-WT clade, the genetic diversity was relatively low (304/2,289 SNPs), whereas isolates outside the clade displayed >20,000 SNPs compared with the most related isolate. Isolate v186-48 was most closely related to the non-WT clade (according to 19,365 SNPs). The remaining 5-FC non-WT isolates, which were not located in this clade, did not cluster. To assess the global dispersal of the resistant clade, we compared the 5 isolates from the Netherlands belonging to the 5-FC non-WT clade, together with the other isolates from the Netherlands (from the WGS data), with 27 previously reported *C. tropicalis* MLST clades. Using WGS SNP analysis, including 1 representative isolate from each MLST clade, we found five 5-FC non-WT isolates from the Netherlands were most closely related to *C. tropicalis* MSLT 15 (Figure 4). The other isolates from the Netherlands formed a distinct branch.

### Molecular Mechanisms of 5-FC Resistance

To investigate the mechanism of 5-FC resistance, we sequenced the *URA3* gene of 30 isolates (6 WT and 24 non-WT) and inspected it for substitutions. The *URA3^K177E^* mutation occurred in all 5-FC non-WT isolates. However, two 5-FC susceptible isolates (M.040-37 and v252-37) also exhibited this mutation (either heterozygous or homozygous mutation), which we confirmed by visual inspection of WGS reads (Appendix Table 4). Therefore, we analyzed the resistance-associated genes *FUR1*, *FCY1*, *FCY2*, and *URA3* by using available WGS data. In *FCY2*, the mutation causing an E49X amino acid nonsense substitution was homozygous in all isolates from the non-WT clade, whereas that mutation was either absent or heterozygous in isolates outside the clade (*40*). For the other resistance-associated genes, no missense mutations were exclusively present in the non-WT isolates. Subsequently, we assessed the isolates for copy number variation by using YMAP (*23*) and compared them with the MYA-3404 reference genome. For *FUR1*, *FCY1*, *FCY2*, and *URA3*, we found no copy number variation in the non-WT clade when compared with the 5-FC–susceptible isolates.

### Epidemiology of 5-FC Non-WT clade

**Figure 5:**
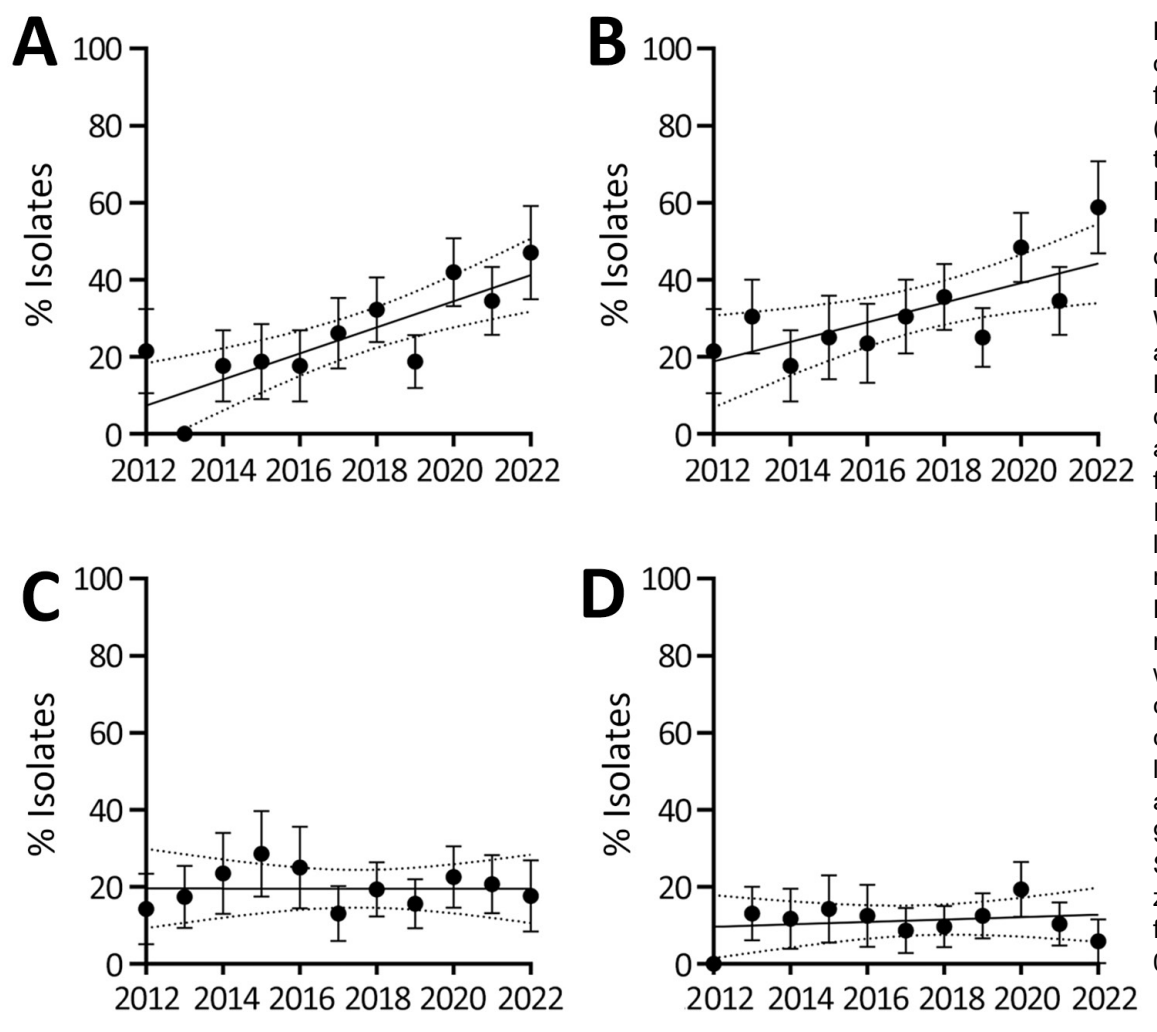
Trends in percentages of flucytosine-resistant and fluconazole-resistant non–wild-type (WT) *Candida tropicalis* isolates, the Netherlands, 2012–2022. A) Percentages of flucytosine (5-FC) non-WT isolates within the specific clade identified in the Netherlands. B) Percentages of all 5-FC non-WT isolates, regardless of clade affiliation. Isolates with a 5-FC MIC above the local epidemiologic cutoff of 0.5 mg/L were classified as non-WT. C) Percentage of fluconazole non-WT isolates. Isolates with a MIC above the local epidemiologic cutoff of 1 mg/L were classified as non-WT. D) Percentage of fluconazole-resistant non-WT isolates. Isolates with values above the EUCAST clinical breakpoint of 4 mg/L were classified as resistant. Solid vertical lines indicate slopes; dotted lines above and below the slope indicate 95% CIs. Error bars indicate SEM. Slopes significantly deviated from zero in panels A and B (p<0.001 for both) but not in panels C (p = 0.992) or D (p = 0.640).

To elucidate the emergence of 5-FC non-WT *C. tropicalis* isolates over time, we compared those isolates with fluconazole non-WT and fluconazole-resistant isolates collected during 2012–2022 (Figure 5). The percentage and clustering of 5-FC–resistant non-WT isolates both increased during this timeframe, particularly since 2018; a substantial peak indicating a higher prevalence in the non-WT population occurred in 2022. In contrast, we observed no downward or upward trend for the fluconazole non-WT or resistant isolates.

## Discussion

We conducted a comprehensive analysis of 5-FC resistance in and genetic relatedness of clinical *C. tropicalis* isolates collected in the Netherlands during 2012–2022. The analyses showed a recent and substantial emergence of non-WT, 5-FC–resistant *C. tropicalis* isolates across the Netherlands since the 2010s. STR typing and WGS identified a circulating non-WT 5-FC–resistant clade; a marked increase in the percentage of isolates belonging to this clade was observed, particularly since 2018.

Approximately one third (32.4%) of the *C. tropicalis* isolates in our collection comprised non-WT strains resistant to 5-FC with a bimodal MIC distribution, indicating a heterogeneous population. Most 5-FC–resistant non-WT isolates had a MIC of 32 mg/L (Figure 1). STR genotyping demonstrated that ≈80% of non-WT isolates were genetically related and formed a distinct clade. The genetic diversity within that clade was low (304–2,289 SNP differences) but distinct, indicating past diversification and environmental spread rather than recent clonal transmission. The increasing prevalence of this clade suggests better adaptation compared with other *C. tropicalis* strains in the Netherlands, although changes in clinical practices and referral patterns might confound this observation.

Our study raises questions regarding the relationship between the 5-FC non-WT clade identified in the Netherlands and similar clades reported in other countries of Europe. A comparison of our WGS data with those of publicly available *C. tropicalis* isolates indicated the clade from the Netherlands is closely related to *C. tropicalis* MLST clade 15 from Denmark (*41*,*42*). A previous global study of 1,571 *C. tropicalis* isolates identified 10 isolates from MLST clade 15 (*42*), which included isolates from Denmark (n = 1) (*41*), Ireland (n = 1) (*43*), United Kingdom (n = 2), Belgium (n = 2), Taiwan (n = 1), and Thailand (n = 3) (*42*). The isolates from Belgium, cultured in 1993 and 1994, had 5-FC MICs of 128 mg/L. The UK isolates, cultured in 2004, had 5-FC MICs of 32 and 64 mg/L. The 5-FC MICs of the isolate from Taiwan cultured in 2006 and those from Denmark (date unknown) and Ireland (cultured in 2018) remain unknown (*41*,*43*). Several isolates from this study clustered with isolates from Belgium and the United Kingdom that had increased 5-FC MICs, indicating spread across Europe (*39*). The MLST 15 isolates from Thailand, cultured during 2015–2017, had 5-FC MICs of <0.5 mg/L (*44*), suggesting intraclade variation in 5-FC susceptibility.

In this study, we analyzed *C. tropicalis* isolates collected during 2012–2022. We conducted an earlier study, which included isolates cultured before 2012 (*17*). In that study, all 18 isolates tested during 2008–2010 were phenotypically 5-FC WT. In 2011, a total of 24 isolates were tested, 6 of which were 5-FC–resistant non-WT isolates. However, genetic analysis of those isolates has not been performed. All isolates collected since 2012 have been genotyped, and the first non-WT isolate from the clade in the Netherlands was identified in 2012 (Figure 5). In parallel with our study, investigators in Denmark also showed a marked increase in 5-FC–resistant, non-WT *C. tropicalis* isolates since the 2010s (*40*). In contrast, 5-FC–resistant non-WT isolates were documented in France as early as the 1980s (*18*), suggesting a later introduction or evolution of the 5-FC–resistant, non-WT clade in the Netherlands. Previous studies using MLST in the United Kingdom, Belgium, and France have also indicated the presence of non-WT *C. tropicalis* clades (*18*,*39*), although the lack of full-genome sequences prevents direct comparisons with our findings.

No 5-FC–resistant, non-WT clade has been reported outside Europe. However, *C. tropicalis* has demonstrated the ability to spread globally, evidenced by the worldwide distribution of other *C. tropicalis* clades. Azole-resistant *C. tropicalis* clades have been documented worldwide (*42*). The fluconazole-resistant MLST clade 4, comprising 248 of 1,571 isolates, mainly originated from Asia, whereas fluconazole-resistant MLST clades 2 and N2 have a global distribution (*42*). Despite the confinement of 5-FC–resistant, non-WT *C tropicalis* to Europe, the widespread prevalence of MLST clades 2 and N2 suggests a potential for global dissemination, emphasizing the importance of vigilance and global surveillance.

We found that 32.4% of *C. tropicalis* isolates were 5-FC–resistant non-WT strains, which was higher than the 19% fluconazole-resistant non-WT and 11% fluconazole-resistant strains. Higher fluconazole resistance rates have been reported in lower and middle income countries, such as China (23.1%) (*42*), Algeria (31.6%) (*45*) and Egypt (37.5%) (*19*), where fluconazole is the primary treatment for invasive fungal infections. Most fluconazole-resistant isolates in our study were from the 5-FC WT population; only 2 isolates exhibited non-WT 5-FC MICs. Despite the rising rate of 5-FC–resistant non-WT isolates, a major national trend was not observed for rates of fluconazole-resistant non-WT isolates.

The factors driving the recent emergence of the 5-FC–resistant, non-WT clade in the Netherlands are unclear. Possible reasons are selective pressure from antifungal drugs or cancer treatments, such as 5-fluorouracil, and better adaptation to human hosts, leading to greater colonization and spread. Unlike azole resistance, which might be linked to extensive clinical or agricultural azole use, 5-FC is rarely used outside medical contexts and seldom prescribed for invasive candidiasis, making resistance development through drug exposure unlikely. Increased 5-FC MICs were not linked to resistance to other antifungal agents, such as fluconazole or echinocandins, and genotyping did not suggest a clonal outbreak. Therefore, the factors behind the rise of this 5-FC–resistant, non-WT *C. tropicalis* clade remain unknown, necessitating further investigation to elucidate mechanisms and prevent spread.

Although 5-FC resistance has been previously associated with the K177E amino acid substitution from a mutation in *URA3*, this association has not been confirmed through transformation studies (*18*). In our study, the K177E mutation was present in all sequenced 5-FC–resistant, non-WT clade isolates; however, it was also detected in non-WT isolates, indicating that this mutation alone cannot fully explain the non-WT resistant phenotype. In Denmark, a mutation in the *FCY2* gene resulting in the E49X amino acid substitution was found in all 5-FC non-WT isolates, possibly overlooked previously because of an error in the *FCY2* reference sequence (*40*). When checking for this mutation, we found it to be homozygous in the 5-FC clade isolates from the Netherlands, which has previously been shown to cause a 5-FC non-WT phenotype (*34*). That laboratory study exposed isolates heterozygous for this mutation to 5-FC and showed that those isolates developed a non-WT 5-FC–resistant phenotype because of loss of heterozygosity for that mutation (*34*). In addition, constructed strains homozygous for the truncated protein and for glutamic acid at amino acid position 49 indicated that the homozygous truncated protein caused a non-WT phenotype, whereas isolates with glutamic acid at position 49 were WT (*34*).

In conclusion, our study confirmed the presence of a 5-FC–resistant, non-WT clade in the Netherlands; similar trends were observed in Denmark (*40*), likely because of the same 5-FC non-WT clade. Further prospective studies are required to gain more epidemiologic insights and clarify the effect of this 5-FC non-WT clade on patient outcomes. Our findings highlight the importance of continuous surveillance, advanced genotyping techniques, and comprehensive clinical data collection to prevent the spread of drug-resistant *Candida* spp. 

Appendix Additional information for emergence of flucytosine-resistant *Candida tropicalis* clade, the Netherlands.
