# Supplementary material for: Emergence of Flucytosine-Resistant Candida tropicalis Clade, the Netherlands
Source: Emerg Infect Dis. 2025 Jul;31(7):1354–64. doi: 10.3201/eid3107.241918 (PMC12205441; doi:10.3201/eid3107.241918)
Supplement: Appendix — Additional information for emergence of flucytosine-resistant Candida tropicalis clade, the Netherlands. [file 24-1918-Techapp-s1.pdf]

Article DOI: https://doi.org/10.3201/eid3107.241918
EID cannot ensure accessibility for supplementary materials supplied by authors. Readers who have difficulty accessing supplementary content should contact the authors for assistance.

Emergence of Flucytosine-Resistant *Candida tropicalis* Clade, the Netherlands

Appendix

Appendix Table 1. Overview of 27 *Candida tropicalis* control isolates*

Identification no.	Country	MLST clade	SRA accession no.
10–03–15–77	the Netherlands	1	SRR18971069
19–008–0062	Australia	2	SRR20760411
ct46	USA	3	SRR12823739
19–008–0006	Singapore	4	SRR20760416
19–008–0078	Australia	5	SRR20760394
10HX058	People’s Republic of China	6	SRR23915243
16GH555	People’s Republic of China	7	SRR23915266
19–008–0034	Australia	8	SRR20760366
ct16	Spain	9	SRR12823772
16JD132	People’s Republic of China	10	SRR23915412
ct05	Ireland	11	SRR12823744
15JS389	People’s Republic of China	12	SRR23915654
10–03–01–41	The Netherlands	13	SRR18971064
SAMEA7387915	Denmark	15	ERR4669790
19–008–0070	Australia	16	SRR20760402
SP4694	Canada	17	SRR11235419
ct67	USA	19	SRR12823716
14TJ531	People’s Republic of China	23	SRR23915558
ct34	Spain	24	SRR12823752
19–008–0018	Singapore	N1	SRR20760386
15FJ462	People’s Republic of China	N2	SRR23915248
SAMEA7387881	Denmark	N3	ERR4669756
11XY004	People’s Republic of China	N4	SRR23915557
14ZZ140	People’s Republic of China	N5	SRR23915557
ct77	Ireland	N6	SRR12823705
ct12	Colombia	N7	SRR12823776
16SJ251	People’s Republic of China	N8	SRR23915614

*Isolates were retrieved from the NCBI SRA database (https://www.ncbi.nlm.nih.gov/sra) along with country and MLST clade number according to reference (*1*). MLST, multilocus sequence typing; SRA, sequence read archive.

Appendix Table 2. Frequency distribution of specimens*

	No. specimens collected			
Anatomic site	5-FC WT	5-FC non-WT non-clade	5-FC non-WT clade	Total
Blood	9	0	5	14
Other sterile site	27	1	20	48
Oropharynx, including sputum/BAL	4	0	2	6
Vagina	1	0	0	1
Feces	5	0	0	5
Urine	10	3	3	16
Other superficial source	12	0	2	14
Unknown	101	12	33	146
Total	169	16	65	250

*Isolates were assigned to clades according to short tandem repeat genotypes (see Appendix Figure). Genotypes 82–150 were allocated to the non-WT clade along with other remaining non-WT isolate. BAL, bronchoalveolar lavage; 5-FC, 5-fluorocytosine; WT, wild-type.

Appendix Table 3. Overview of antifungal MICs of 250 *Candida tropicalis* isolates according to EUCAST broth microdilution*

Antifungal agent	MIC range, mg/L	GM	MIC_{50}, mg/L	MIC_{90}, mg/L
5-FC, n = 250	0.063–64	0.578	0.125	32
AMB, n = 249	0.063–1	0.398	0.5	1
FLU, n = 247	0.063–≥64	0.757	0.5	8
ITC, n = 97	≤0.016 to ≥64	0.040	0.031	0.125
VOR, n = 249	≤0.016 to ≥8	0.048	0.031	0.25
POS, n = 52	≤0.016–0.25	0.030	0.031	0.063
MICO, n = 199	≤0.016–4	0.098	0.063	0.5
AFG, n = 246	0.008–0.125	0.017	0.016	0.031
CAS, n = 176	0.125–1	0.353	0.5	0.5
MFG, n = 198	0.004–0.063	0.014	0.016	0.031

*AMB, amphotericin B; AFG, anidulafungin; CAS, caspofungin; 5-FC, flucytosine; FLU, fluconazole; GM, geometric mean; ITC, itraconazole; MFG, micafungin; MICO, miconazole; POS, posaconazole; VOR, voriconazole.

Appendix Table 4. Overview of the *URA3* genotype of 30 *Candida tropicalis* isolates categorized as WT and non-WT*

Identification no.	Category	*URA3*
M.010–14	non-WT	K177E
M.019–30	WT	WT
M.022–48	non-WT	K177E
M.025–18	non-WT	K177E
M.031–29	non-WT	K177E
M.034–69	non-WT	K177E
M.040–37	WT	K177E
M.050–60	non-WT	K177E
M.051–35	non-WT	K177E
M.053–22	non-WT	K177E
v178–35	non-WT	K177E
v189–63	non-WT	K177E
v200–30	WT	WT
v213–13	non-WT	K177E
v230–50	non-WT	K177E
v234–60	non-WT	K177E
v239–71	non-WT	K177E
v251–81	non-WT	K177E
v252–37	WT	K177E†
v261–74	non-WT	K177E
v262–70	non-WT	K177E
v269–61	non-WT	K177E
v277–77	non-WT	K177E
v307–69	WT	WT
V308–10	WT	WT
v308–71	non-WT	K177E
v310–76	non-WT	K177E
v314–20	non-WT	K177E
v318–62	non-WT	K177E
v318–81	non-WT	K177E

*Isolates with a MIC ≤0.5 mg/L were classified as WT and those with a MIC >0.5 mg/L as non-WT. WT, wild-type.
†Mutation is heterozygous.

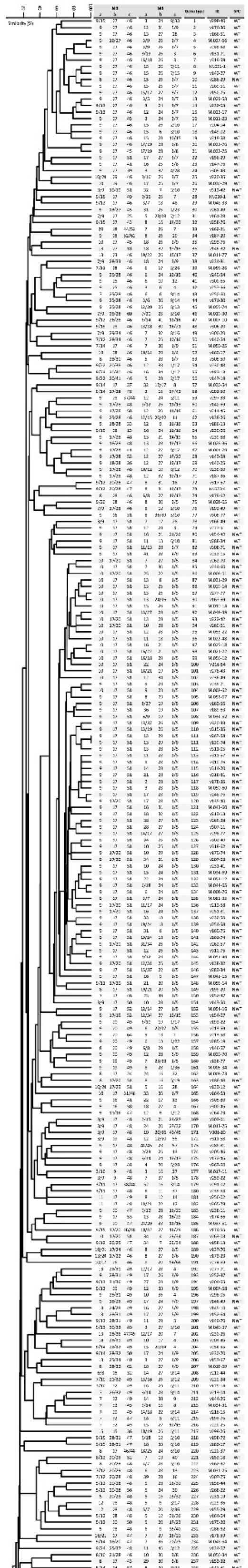

Appendix Figure. Cluster analysis of 250 *Candida tropicalis* isolates. Branch lengths indicate relatedness according to microsatellite alleles. Isolates with a MIC of ≤0.5 mg/L were classified as susceptible and >0.5 mg/L as resistant.

References

1. Fan X, Dai RC, Zhang S, Geng YY, Kang M, Guo DW, et al. Tandem gene duplications contributed to high-level azole resistance in a rapidly expanding *Candida tropicalis* population. Nat Commun. 2023;14:8369. PubMed https://doi.org/10.1038/s41467-023-43380-2
